# A Population-Based Study of Meconium Aspiration Syndrome in Neonates Born between 37 and 43 Weeks of Gestation

**DOI:** 10.1155/2012/321545

**Published:** 2011-11-30

**Authors:** C. Fischer, C. Rybakowski, C. Ferdynus, P. Sagot, J. B. Gouyon

**Affiliations:** ^1^Department of Pediatrics, CHU Dijon, 21000 Dijon, Burgundy, France; ^2^Department of Medical Informatics and Biostatistics, CHU Dijon, 21000 Dijon, Burgundy, France; ^3^Departement of Obstetrics, CHU Dijon, 21000 Dijon, Burgundy, France

## Abstract

The epidemiology of meconium aspiration syndrome (MAS) in term neonates is described in a population-based retrospective study of data recorded for all births from 2000 to 2007 in a French region (Burgundy). Of the 132 884 eligible term newborns, the rate of meconium-stained amniotic fluid (MSAF) was 7.93%. The prevalence of severe MAS was 0.067% in the overall population. MAS rate was 0.11% at 37-38 weeks of gestation (WG), 0.20% at 39–41 WG, and 0.49% at 42-43 WG. Factors independently associated with severe MAS were identified by a case-control study, that is, thick meconium amniotic fluid, fetal tachycardia, Apgar score ≤3 at 1 minute, and birth in a level III facility. Our results confirm the high prevalence of MSAF after 37 WG but also show the low frequency of severe MAS in a period corresponding to the new international recommendations on the management of birth with MSAF.

## 1. Introduction

Meconium aspiration syndrome (MAS) is an infrequent but life-threatening respiratory disease affecting some of the infants born through meconium-stained amniotic fluid (MSAF).

MAS may be a severe condition as 30 to 50% of MAS required mechanical ventilation or continuous positive airway pressure (CPAP) [1, 2].

MAS is frequently associated with fetal hypoxia which promotes meconium discharge in amniotic fluid, gasping and aspiration of MSAF, and also changes in the vascular muscular media of pulmonary arteries of the fetus [3, 4].

In the early 2000, the prevalence of MAS ranged from 0.20% to 0.54% in the general population [5–7] and from 1.0% to 6.8% in infants born through MSAF [3, 5–7]. A review of ten reports published from 1990 to 1998 showed a combined incidence of 13.1% for MSAF, 0.52% of MAS, 4.2% of MAS among MSAF, and 49.7% of MAS requiring ventilatory support with a 4.6% mortality rate [6].

However, large population-based studies were scarce and suggested a lower incidence of MAS: the national US birth cohort study conducted on the basis of singleton term non-Hispanic white live births (1995–2001) showed that the rate of MAS markedly increased with gestational age (GA), that is, from 0.10% at 37 weeks gestation (WG) to 0.22 and 0.31% at 40 and 41 WG, respectively [8]. The prevalence of MAS could be extrapolated to 0.18% in this population of term infants. In Australia, the rate of MAS requiring mechanical ventilation in level III units ranged between 0.024 to 0.046% at 36–40 WG and then increased to 0.080% at 41 WG and 0.14% at 42 WG [9]. In France, the prevalence of mechanically ventilated MAS was estimated to 0.043% by a retrospective national survey among neonates born in 2000-2001 [10].

Previous studies identified several risk factors of MAS that is, fetal compromise indicated by abnormalities of fetal heart rate tracings and/or poor Apgar score [1, 11–16] and/or low cord pH [5, 17]; Cesarean delivery [1, 18]; ethnicity (black Americans, Africans, Pacific Islanders); advanced gestation [15, 19, 20]. However, studies based on the global population did not specifically address the determination of risk factors of severe MAS among infants born through MSAF. It is worthy to note that regionalization of perinatal care concentrated high risk pregnancies in level III facilities, a condition representing a potential bias in assessing prevalence of severe MAS and identification of risk factors.

Therefore, a population-based study was designed to confirm the low prevalence of severe MAS and identify risk factors of severe MAS among term infants born in MSAF.

## 2. Methods

The database of the Burgundy perinatal network was studied for the years 2000–2007. The Burgundy perinatal database is a voluntary collaboration between all 18 public and private hospitals in Burgundy (level III: 1; level II: 7; level I: 10) [21, 22]. The database which was set up with the approval of the National Committee of Informatics and Liberty includes all mother-infant pairs. Perinatal data are recorded at the time of neonatal discharge from maternities or neonatal units. Procedures are established to ensure quality of the recorded data, including standardized definitions, guidelines for coding, and validation of data coherence by specific softwares [21, 22].

All live births are eligible if their GA was ≥37 WG' estimated GA. Exclusion criteria included severe congenital malformations, chromosomal abnormalities, congenital neuromuscular diseases, and metabolic diseases.

All cases of MSAF and MAS were identified in the perinatal database. Severe MAS (i.e., treated by mechanical ventilation and/or continuous positive airway pressure) and their controls were confirmed when the medical records were systematically reviewed and abstracted by an independent neonatologist assessor (C.R or C.F). The diagnosis of MAS was established according to diagnostic criteria from Rubaltelli et al. [23], that is, respiratory distress with elevated oxygen dependence; presence of meconium in amniotic fluid; chest radiograms with massive bilateral patchy infiltrates with or without pleural fluid. For each patient, the type MSAF was qualified as “thin” when the fluid was just tinted yellowish or slightly greenish, “moderate” when it was really greenish, but fluid and “thick” when it was green and thick.

The GA, in completed weeks, was assessed on the basis of the mother's last menstrual period as confirmed or modified where necessary by routine early antenatal ultrasound examination. Owing to the low prevalence of MAS, a case/control study appeared optimal. For each case of severe MAS, 3 controls born in MSAF without any respiratory distress at birth were obtained from the regional database. Cases and controls were paired according to GA assuming a preponderant role of GA in MAS incidence [8, 9, 24–28]. The recorded variables are those shown in Table 2. Fetal heart rate recordings were precisely reviewed and classified according to French guidelines (fetal tachycardia, bradycardia, decelerations, decreased variability) [29].

### 2.1. Statistics

Quantitative data were presented as a mean and standard deviation (SD), and compared by one-way analysis of variance or, if normality or homoscedasticity assumptions, were violated Mann-Whitney *U*-test. Qualitative data were presented as percentages and compared using Pearson Chi Square or, in the case of very rare conditions, Fisher's exact test.

Secondly, a conditional logistic regression was used to determine significant independent variables associated with an increased risk of severe MAS. The model was adjusted for amniotic fluid, Apgar score ≤3 at 1 min, level of birth place, mode of delivery, insufficient followup during pregnancy, and tachycardia or bradycardia on FHR recordings. A *P* value below 0.05, for a 2-tailed Wald test, was considered as statistically significant. First-order interactions were systematically tested by a 2-tailed Wald test and excluded from the model if they did not reach statistical significance at the 0.05 level. Adjusted odds ratio (OR) and their 95% confidence intervals (CI) were calculated.

Statistical analyses were performed using SAS 9.2 (SAS Institute Inc) and Stata 8.0 (StataCorp LP) packages.

## 3. Results

The Burgundy perinatal database collected a total of 133 087 births with GA ≥37 WG from the period beginning January 1st, 2000 and ending on December 31st, 2007. Of these births 203 had exclusion criteria and 10 540 of the 132 884 eligible neonates (7.93%) were delivered within MSAF (thin, moderate, or thick).

**Figure 1:**
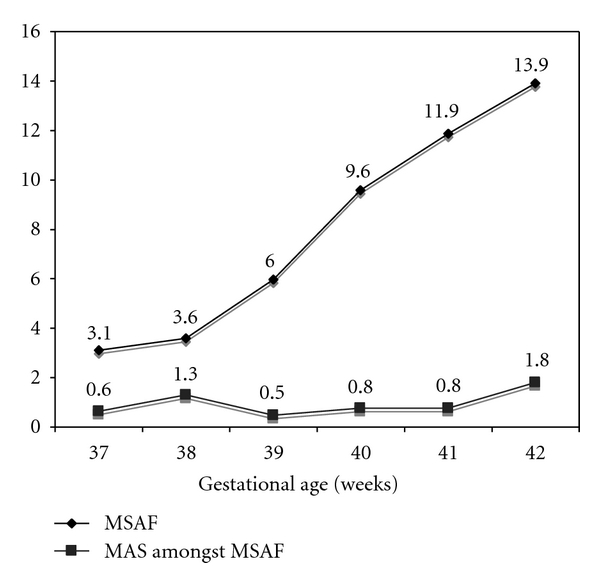
Rates of meconium-stained amniotic fluid (MSAF) in the global population and rate of meconium aspiration syndrome (MAS) amongst MSAF according to gestational age in term and postterm deliveries.

The incidence of MSAF linearly increased with GA (Figure 1). The rate of MSAF was 3.52% at 37-38 WG versus 9.07% at 39–41 WG (OR = 2.74 [2.56 to 2.92, *P* < 0.0001]) and 14.37% at 42-43 WG (OR = 4.60 [4.03 to 5.26; *P* < 0.001]).

**Figure 2:**
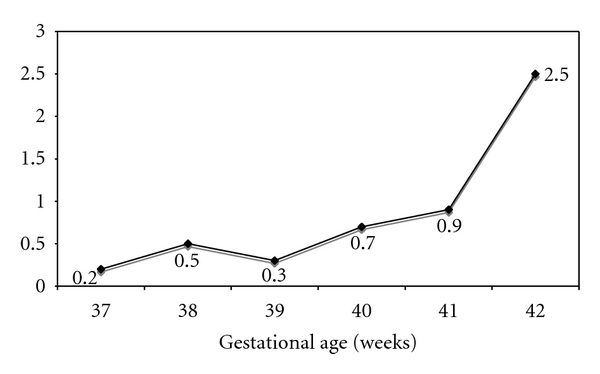
Rates of meconium aspiration syndrome (MAS) in the global population according to gestational age in term and post-term deliveries.

The regional database identified 241 neonates with MAS. The prevalence of MAS were 0.18% of the overall population. The incidence of MAS markedly increased with GA after 39 WG (Figure 2). The rates of MAS were 0.11% at 37-38 wks versus 0.20% at 39–41 wks (OR = 1.86 [1.27 to 2.72, *P* = 0.0013]) and 0.49% at 42-43 wks (OR = 4.65 [2.34 to 9.27; *P* < 0.0001]).

The odds ratios of MSAF and MAS at each gestational age are indicated in Table 1.

The global prevalence of MAS in neonates born with MSAF was 2.29% and did not significantly change with GA (Figure 1).

Among the 241 MAS, treatment modalities were oxygen alone in 152 (63.1%), nasal CPAP without mechanical ventilation in 3 (1.2%), conventional ventilation without high frequency oscillation (HFO) in 69 (28.6%), HFO in 17 (7.1%). Therefore, severe MAS (i.e., MAS treated by mechanical ventilation and/or nasal CPAP) was identified in 89 neonates. The prevalence of severe MAS was 0.067% of the overall population, 0.84% of neonates born through MSAF, and 36.9% of MAS.

Amongst the 89 severe MAS the median of duration of mechanical ventilation (conventional ventilation and HFO) was 2.0 days (1.0–5.0). Surfactant was given to 34 and antibiotics to 79 of the 89 infants with severe MAS (38.2% and 88.9%, resp.). Nitric oxide inhalation and ECMO were used in 15 (16.8%) and 2 (2.2%) infants, respectively. The neonatal course was associated with persistent pulmonary hypertension of the neonate in 14 (15.7%), air leaks in 10 (11.4%), hypotension in 20 (22.5%), and late-onset neonatal infection in 4 (4.5%). Concerning thin versus moderate or thick meconium, mechanical ventilation duration was 1.0 (1.0–4.0) versus 2.0 (1.0–6.0; *P* = 0.30) days and death rate was 6.7% versus 8.5% (*P* = 0.76). There were 7 deaths (7.9%) of which 4 were related to severe ischemic encephalopathy and 3 were due to intractable pulmonary haemorrhage, sepsis, and multiple organ failure.

The 89 neonates with severe MAS were paired to 267 controls according to GA (40.0±1.2 weeks). Gestational age was 43 weeks in 6.7% of the neonates in this case-control study. Mean birthweight was 3388 ± 549 g in cases and 3329 ± 476 g in controls (*P* = 0.33).

Bivariate analysis identified significant risk factors associated to severe MAS (Table 2), that is, gestation with insufficient followup; birth place in a level III facility; delivery during day time; moderate and thick meconium; amnioinfusion; fetal tachycardia and fetal bradycardia; CS delivery; low Apgar score at 1 and 5 min; tracheal aspiration at birth and pediatrician intervention at birth.

The conditional logistic regression analysis identified independent variables significantly associated with an increased risk of severe MAS: amniotic fluid stained by moderate or thick meconium versus thin meconium (OR = 5.63 [2.52 to 12.6; *P* < 0.0001]); fetal tachycardia, (OR = 4.17 [1.27 to 3.7; *P* = 0.019]); Apgar score ≤3 at 1 min, (OR = 87.5 [18.9 to 405; *P* < 0.0001]); or birth in the level III facility in comparison with birth in the level II facilities (OR = 4.8 [2.0 to 11.6; *P* = 0.0005]).

## 4. Discussion

In this epidemiological study, MSAF is a frequent event accounting for 7.9% of all deliveries in non-preterm neonates. On the other hand, MAS is a rare event (0.18%) with a need for mechanical ventilation and/or nasal CPAP in approximately one-third of them as reported in other studies [1, 2, 6]. Avoiding mechanical ventilation by using nasal CPAP has probably a marginal role as only 1.2% of MAS are treated with this ventilatory mode. Goldsmith [2] recently highlighted that the optimum modes of ventilation for MAS are not known. He pointed out that high-frequency ventilation, inhaled nitric oxide, surfactant and extracorporeal membrane oxygenation are rarely required. Our series confirms this observation as only 12.0% of MAS required those treatments.

The overall incidence of MAS and severe MAS increases with GA as reported in recent population-based studies [8, 9]. The overall rates of MAS in the USA [8] and Burgundy are similar: 1.0 versus 1.1 per 1000 live births (‰) at 37 WG; 1.1 versus 1.0‰ at 38 WG; 1.5 versus 1.1‰  at 39 WG; 2.2 versus 2.4‰  at 40 WG, and 3.1 versus 2.6‰  at 41 WG. Furthermore the incidence of severe MAS recorded in Australia [9] at 41 WG (0.80‰) is close to the 0.67‰  observed at 39–41 WG in our series. So, our cohort of MAS can be regarded as representative of this pulmonary disease in the 2000 s in developed countries. It is interesting to note that the recent population based studies [8, 9] showed a low prevalence of MAS as compared to monocentric and multicentric studies usually conducted in level III facilities. Our series confirms the bias that could be induced by nonepidemiological studies of MAS incidence as it shows that birth in a level III facility is an independent risk factor of severe MAS. It can be speculated that regionalization of perinatal care concentrates high risk pregnancies in level III facilities and increases the risk for MAS. Finally, the case-control study suggested that the care in the Burgundy population with MSAF was characterized by low rates of amnioinfusion, obstetrical naso-oropharyngeal aspiration and tracheal aspiration in infants born with MSAF but without MAS. This fits well with current recommendations [30] about management of infants born through MSAF: oronasopharyngeal suctioning before the delivery of the shoulders in infants born through MSAF is useless in the prevention of MAS; tracheal suctioning should be selectively applied to nonvigorous neonates born in MSAF according to the pivotal study of Wiswell et al. [31].

In this study the incidence of MAS is stable from 37 to 39 WG and increases afterwards particularly in infants born at 42-43 WG: the risk of MAS is approximately 4-fold and 27-fold at 42 WG and 43 WG in comparison to 37 WG.

However, the incidence of MAS in neonates born through MSAF does not vary significantly with GA. Similarly, population-based studies as well as nonpopulation-based studies showed that prolonged pregnancy (≥42 weeks) increases perinatal morbidity and mortality and greatly favours MSAF and MAS [7, 30, 32]. 

Some studies suggested that prevention of postterm pregnancy prevents severe MAS [33]. The earlier induction of labour (e.g., by 41 weeks) may prove to be beneficial for the prevention of the MAS as shown by Ross [34]. A recent Cochrane review [26] shows that a policy of labour induction is associated with a reduced incidence of MAS at both 41 completed WG (relative risk (RR) = 0.29 [0.12 to 0.68]) and 42 completed WG (RR = 0.66 [0.24 to 1.81]). However, a new randomised clinical trial [35] does not found any significant differences between induced and monitored postterm neonates regarding neonatal morbidity. New prospective randomised studies are required to establish whether labour induction reduces MAS incidence without promoting other respiratory diseases such as transient tachypnea of the newborn (TTN) or respiratory distress syndrome (RDS).

The low incidence of severe MAS and the preeminent role of GA on this incidence justified a case-control study [36] paired on GA. Amniotic fluid stained by moderately or markedly thick meconium, fetal tachycardia, and Apgar score ≤3 at 1 min promote severe MAS. Thick meconium is usually regarded as a common finding in severe meconium aspiration syndrome [3, 6, 19, 37], and most studies were focused on neonates born through moderate or thick MSAF. However, a lack of correlation between the severity of MAS and the thickness of meconium has been previously suggested by Ghidini and Spong [25] and Suresh and Sarkar [38]. Our series confirms this hypothesis as thick meconium is found in only 11% of severe MAS (1% of controls) while moderate and thin meconium concern 55% and 34% of severe MAS, respectively. Apgar score ≤3 at 1 min is the main risk factor as the relative risk is 87. Low Apgar score has been universally associated with MAS [1, 3, 6, 9, 11–16, 28, 32, 39, 40], and the role of fetal hypoxia has also been ascertained by elevated cord blood concentrations of erythropoietin in both MSAF [41] and MAS [32, 42].

It is well known that poor antenatal conditions are predominant in determining MAS. This point of view is reinforced as fetal tachycardia is another independent risk factor of severe MAS in this study. This observation has been made in many other studies, not reassuring fetal heart rate monitoring being widely associated with MAS [1, 11–16, 18, 43, 44]. However, contradicting data were obtained by Mitchell et al. [45] who concluded that FHR tracings are relatively poor predictors of the presence of fetal acidosis when amniotic fluid is meconium stained.

These overall results suggest that severe MAS is an antenatal disease thus justifying adapted antenatal care. Nowadays, identification of perinatal asphyxia remains a major endpoint of MAS prevention [33]. Guidelines of earlier fetal monitoring (e.g., by 40 WG) proved to be beneficial for the prevention of MAS [34].

Finally, our series failed to identify risk factors reported in some other studies, that is, nulliparity [32, 37], male gender [3], previous Cesarean section [16], and oligohydramnios [3]. Although our work is a population-based study in an entire French region with 1.8 millions inhabitants, there are only 89 cases of severe MAS over an 8-year period. A low sample size may have favored the low number of factors significantly associated with severe MAS.

## 5. Conclusion

Our series confirms the high prevalence of MSAF after 37 WG but also shows the low incidence of both MAS and severe MAS in a period corresponding to the implementation of the new international recommendations on the management of neonates born in MSAF [30].

The main risk factor of MSAF is GA but the incidence of MAS in neonates born in MSAF does not depend on GA. Our series indicates that moderate or thick amniotic fluid and fetal tachycardia may help to anticipate the need for neonatal resuscitation in delivery room whatever is GA. Further studies comparing perinatal factors associated with severe and nonsevere MAS could be useful to help clinicians in delivery room to anticipate severe MAS, a rare event which remains life threatening.

## Figures and Tables

**Table 1:** Odds ratios (95% CI; *P* value) of meconium-stained amniotic fluid (MSAF) and meconium aspiration syndrome (MAS) in neonates born between 38 and 43 WG compared to neonates born at 37 WG.

	38 WG	39 WG	40 WG	41 WG	42 SA	43 WG
MSAF	1.11 [0.97, 1.28; *P* = 0.13]	1.94 [1.71, 2.20; *P* < 0.0001]	3.22 [2.84, 3.63; *P* < 0.0001]	4.08 [3.61, 4.62; *P* < 0.0001]	4.96 [4.20, 5.87; *P* < 0.0001]	5.29 [2.03, 13.8; *P* = 0.0007]

MAS	0.89 [0.42, 1.90; *P* = 0.77]	0.96 [0.48, 1.93; *P* = 0.92]	2.10 [1.09, 4.03; *P* = 0.026]	2.25 [1.16, 4.38; *P* = 0.016]	3.97 [1.65, 9.55; *P* = 0.002]	27.3 [3.40, 220; *P* = 0.002]

**Table 2:** Case-control comparison in a population of neonates with GA ≥37 weeks and born through MSAF. Cases are severe MAS (i.e., treated by mechanical ventilation and/or continuous positive airway pressure). Paired neonates without respiratory symptoms are controls.

	Cases *n* = 89	Controls *n* = 267	*P* value
Characteristics of the mother			
Age (years)	29.7 ± 5.8	28.9 ± 5.2	0.23
Nulliparity (%)	59.5	58.1	0.80
Past history of CS^a^ (%)	7.95	7.87	0.97

Characteristics of pregnancy			
Multiple pregnancy (%)	0	1.5	0.24
Insufficient followup care (%)	8.99	0.75	<0.0001
Smoking (%)	21.6	18.5	0.52
Hypertension or preeclampsia (%)	2.28	4.91	0.28
Oligohydramnios (%)	4.5	3.4	0.62
Antenatal diagnosis of IUGR^b^ (%)	1.12	2.64	0.40
Gestational diabetes (%)	2.28	6.0	0.16
Clinical chorioamnionitis (%)	0	0	1.0
GBS^c^ vaginal carriage (%)	12.9	9.4	0.35
Placenta praevia (%)	0	0	1.0
Placental abruption (%)	0	0	1.0

Characteristics of delivery			
Cord abnormalities	23.6	26.7	0.53
PROM^d^ (>12 hr)	4.6	9.7	0.13
Antenatal steroid therapy	2.2	0	0.25

Birth place:			
Level I (%)	16.8	11.2	
Level II (%)	49.5	77.	
Level III (%)	33.7	11.2	<0.0001
Day time delivery (%)	56.2	42.6	0.02
Induction of labor (%)	27.0	28.1	0.83

Meconium in amniotic fluid			<0.0001
Thin (%)	33.7	76.4	
Moderate (%)	55.1	22.8	
Thick (%)	11.2	0.78	
Amnioinfusion (%)	7.9	1.9	0.0067

Fetal Heart Rate (FHR):			
Tachycardia (%)	17.2	4.5	0.0001
Bradycardia (%)	49.4	32.9	0.0057

Presentation:			
Cephalic (%)	95.5	97.4	
Breech (%)	3.4	1.9	0.37
Other (%)	1.1	0.7	

Anesthesia			
(i) Spinal (%)	14.6	9.4	
(ii) Epidural (%)	69.7	67.0	
(iii) General (%)	2.2	3.0	0.29
(iv) No anesthesia (%)	13.5	20.6	

Mode of Delivery			
CS (%)	37.2	20.2	
Vaginal with manoeuvres (%)	17.9	17.9	
Vaginal without manoeuvres (%)	44.9	61.9	0.004
Obstetrical aspiration (%)	5.6	3	

Characteristics of neonates			
Sex ratio (% male)	47.2	48.3	0.85
Mean birthweight (g) (±SD)	3388 (±549)	3329 (±476)	0.33
BW^e^<10th perc. (%)	19.1	13.8	
BW ≥10th perc. ≤ 90th perc. (%)	67.4	77.6	
BW >90th perc. (%)	13.5	8.6	0.15
Apgar at 1 min ≤3 (%)	51.7	1.1	<0.0001
Apgar at 5 min ≤5 (%)	32.5	0.0	<0.0001
Tracheal aspiration at birth (%)	80.9	8.2	<0.0001

First care:			<0.0001
Pediatrician (%)	73.4	28.5	
Midwife (%)	23.6	71.5	

^a^CS: Cesarean section, ^b^IUGR: Intrauterine growth restriction, ^c^GBS: Group B Streptococcus, ^d^PROM: prolonged rupture of membranes (>12 hours), ^e^BW: birth weight.
